# Bio-Based Compounds from Grape Seeds: A Biorefinery Approach

**DOI:** 10.3390/molecules23081888

**Published:** 2018-07-28

**Authors:** Massimo Lucarini, Alessandra Durazzo, Annalisa Romani, Margherita Campo, Ginevra Lombardi-Boccia, Francesca Cecchini

**Affiliations:** 1 CREA—Research Centre for Food and Nutrition, 00178 Roma, Italy; alessandra.durazzo@crea.gov.it (A.D.); g.lombardiboccia@crea.gov.it (G.L.-B.); 2 PHYTOLAB, University of Florence, 50019 Sesto Fiorentino, (Firenze), Italy; annalisa.romani@unifi.it (A.R.); margherita.campo@unifi.it (M.C.); 3 CREA—Research Centre for Viticulture and Enology, 00049 Velletri, Roma, Italy; francesca.cecchini@crea.gov.it

**Keywords:** biorefinery, circular economy, grape seed, bio-based, chemometrics

## Abstract

Food and agricultural waste represents a growing problem with negative effects on the economy, environment, and human health. Winemaking produces byproducts with high added value, which can be used for new productions in several application fields. From the perspective of biorefinery and circular economy, grape seeds could be exploited by extracting bioactive compounds with high added value before using biomass for energy purposes. The markets concerned are, in addition to the food, cosmetics, and pharmaceuticals sectors, which use bioactive compounds, the sector of biopolymeric materials and of energy for the production of biohydrogen and biomethane. Generally, bioactive components should be investigated through an integrated and multidisciplinary study approach based on emerging analytical techniques; in this context, attention is addressed towards green and sustainable procedures; an update of extraction techniques, innovative technologies, and chemometrics are described. Nowadays, processes so far tested on a pilot scale for grape waste are developed to enhance the extraction yields. Here, a picture of the Italian experience applied to the byproducts of the wine industry is given.

## 1. Introduction

Food and agricultural waste is a growing problem that, if not properly addressed, has negative effects on the economy, environment, and human health. Food and agricultural waste can not only be avoided but it is possible to intercept the opportunity to exploit the waste to define, for example, new bioproducts in a more complete view of biorefinery. It has been estimated that a third of all the food produced in the world is not consumed, making for a total of about 1.3 billion tons of waste a year [1,2].

In Italy, the economic value of food waste is around 8.5 billion euro per year [3], of which about 11% (equivalent to €180 million) takes place during industrial processing in the fruit and vegetables sector [4].

In Europe, viticulture plays a fundamental role, with a market dominated by Italy, France, and Spain. Vinification is wrongly considered a production with low environmental impact; instead, it requires considerable quantities of resources such as water, soil fertilizers, amendments, producing a huge amount of waste [5,6]. From an agronomic point of view, the vineyard area in Italy is about 5.2% of the Used Agricultural Surface (SAU), equivalent to 664,296 hectares at national level [7], with a wine production of 47 million hectoliters in the 2017 harvest (biggest wine producer by volume) [8,9]. Winemaking produces a series of byproducts, easily exploitable for new productions and in various supply chains.

During the winemaking process, the quality and quantity of these byproducts depend on a set of cofactors, but basically on the type of vinification [5,10,11,12,13]. Grape seeds represent the portion of fruit with the highest concentration of bioactive molecules; several studies reported that, among the different parts of grape fruit, seeds show the highest antioxidant activity, followed by the skin and pulp [14,15].

Grape seeds still have great biological potential that could be exploited by extracting bioactive compounds with high added value before using biomass for energy purposes to obtain extracts and semifinished products useful for agronomic, cosmetics, feed, food, nutraceutical, and pharmaceutical purposes.

Therefore, considering grape seeds as waste represents a double loss for the food industry, both for the disposal costs and for the loss of profits deriving from their recycling and exploitation.

From the processing of one ton of grapes, approximately 0.13 tons of pomace are produced, 0.06 tons of lees, 0.03 tons of pomace, and 1.65 m^3^ of waste water [5,6]. Seeds can be easily recovered from pomace by separation and sifting technologies. Accounting for 8–20% of the weight of grapes processed by the wine industry [11,16], it can be estimated that in Italy there is an amount of seeds coming from the winemaking process that varies between 0.1 and 0.3 million tons [17,18].

The disposal of waste and byproducts led to strong environmental impact that can be estimated by assessing the carbon footprint that is slowly also spreading in the wine sector: the most widely used methodology is the Life Cycle Assessment (LCA) [12,19,20,21,22,23,24,25]; on the Italian level, the calculation of the emission of CO_2_ equivalents due to the 2016 vinification waste was calculated on the basis of the “International Wine Carbon Calculator” [26] and was equal to 278,100 tons from lees, 834,300 tons from pomace, and 185,400 tons from stems [27]; in Italy in the 2016 harvest, 1300 tons of CO_2_ equivalents were produced, which could be reduced by using these byproducts as raw material for other production chains, as underlined by Bevilacqua et al. [27].

The creation of an integrated model with a biorefinery approach applied to the oenological sector would therefore allow considering winemaking waste as co-products in a virtuous circular economy process aligned with European waste legislation and meeting or exceeding the Kyoto protocol goals [28].

## 2. The Biorefinery Concept

A biorefinery is a facility that provides for an integrated, efficient, and flexible conversion of biomass feedstocks through a combination of physical, chemical, biochemical, and thermochemical processes into multiple products. As a broad technological definition, biorefinery is intended as the conversion of all kinds of biomass (e.g., organic residues, energy crops, aquatic biomass) into a wide range of bio-based products, such as food and feed, chemicals, materials, fuels, power, and heat [29,30].

Biorefineries are considered the most complete way for the creation of an industry based on products derived from biological materials or able to enhance the different chemical components of biomass.

The concept of biorefinery is analogous to oil refineries but tends to exceed the limit of a purely energetic crop destination, proposing the use of plant biomass to extract chemicals to be used in several sectors. The biorefinery deals with separating the components of the biomass in its constituents to assign each of them to the use that allows obtaining the greatest yield in terms of biovalue. The biorefinery approach, applied to winemaking, provides for the optimal exploitation of byproducts in industrial sectors not in competition with that of wine.

**Figure 1:**
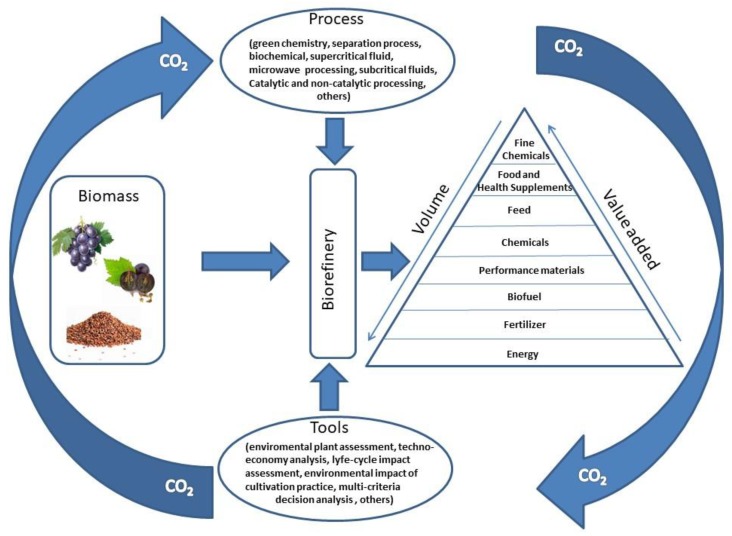
The biorefinery concept: a puzzle piece of circular bioeconomy.

Biorefinery outputs are classified into a biovalor hierarchy that indicates the value of biomass transformations (Figure 1) based on new circular agricultural or economic models. At the top of the pyramid there are substances for fine and pharmaceutical chemistry, useful for the synthesis of vaccines, antibiotics, and immunotherapy proteins. Further down the hierarchy of biorefinery products, there are food and feed products, followed by substances for the chemical industries, such as bioplastics, lubricants, solvents, adhesives, fibers, and dyes. At the bottom of the pyramid there are all the substances for the production of biogas by fermentation and biofuels in the field of energy sector.

The more suitable technologies for separation, fermentation, gasification, and chemical conversion must be identified, as well as for biomasses’ pretreatment and storage.

In addition to territorial integration, favored by the reduced size of the plants compared to those of traditional petrochemicals, the availability of biomass requires the development of integration processes within the relationship with suppliers; this allows access to a limited supply range to qualified materials to be included in the production processes.

In terms of efficiency and sustainability, chemical products from biomass should be considered and certified on the basis of criteria referring to the entire life cycle of products [31]. According to estimates by international organizations, the transition to a green economy could generate from 15 to 60 million jobs (green jobs) globally in the next twenty years [32]. Communication also plays a role of primary importance: informing consumers about the safety aspects, as well as about the benefits that can arise from good planning of the valorization of biomass, will be even more than any other incentive paths [18].

## 3. High Value-Added Compounds in Grape Seed

The waste generated by the agroindustry should be considered for a biorefinery approach, as they meet different criteria, such as the quantity of raw material and the content in molecules with high added value [33,34,35,36]. The interest in grape seeds has increased, especially for their content in nutraceuticals, such as phenolic compounds (gallic acid, hydroxybenzoic and cinnamic acid derivatives, quercetin, kaempferol, monomeric flavan-3-ols, i.e., (+)-catechin, (−)-epicatechin, gallocatechin and epicathechin 3-*O*-gallate, procyanidin dimers, trimers, and more highly polymerized procyanidins) [37], unsaturated fatty acids [38,39,40], vitamin E, carotenoids, and phytosterols [41].

Several studies reported potential protective properties of grape seeds [42,43,44,45,46,47,48], i.e., the anticancer and chemopreventive efficacy of grape seed extract against various types of cancers [49,50,51,52]. The beneficial effects of grape seed on human health are due to the concerted and combined action of bioactive compounds; it is clear that the first step of bioactivity is linked to antioxidant properties [53,54].

Other than genetic traits of the grape cultivar, extrinsic factors as the year of production (i.e., the climatic condition from year to year), the site of production (such as the effect of geographic origin of grapes, soil composition, and fertilization), the degree of maturation, and postharvest practices strongly influence the levels of nutraceutical compounds [55,56,57,58]. Furthermore, processing technologies, as type of vinification and some related steps, e.g., fermentation, represent additional factors in determining the bioactive profile of grape seeds [59,60].

The identification, isolation, and quantification of functional grape seed components, as well as the assessment of their interactions, are necessary prerequisites for selecting adequate procedures in biorefinery. For this reason, particular attention should be paid towards fast and green procedures and alternative analytical techniques.

## 4. Green and Sustainable Procedures: Extraction Techniques, Innovative Technologies, and Chemometrics

Generally, studies on the evaluation of bioactive components should be integrated in a multidisciplinary and innovative study approach for food research: the combination of emerging analytical techniques and the application of statistical methods in food science led to the innovative challenge for modelling agrofood systems.

Sampling and extraction procedures are crucial processes for the recovery, isolation, and identification of bioactive compounds from grape seeds. The optimal extraction procedure should provide the maximum yield in terms of concentration of target compounds. Different variables should be considered—pretreatment of the sample, solvent/sample ratio, type of solvent, particle sizes, time and temperature of extraction, and so on [61,62].

Conventionally, several types of pressing machines and different conventional solvent extraction techniques have been applied in the analysis of bioactive compounds from food wastes. These techniques are generally based on the use of toxic compounds, restricting the application of grape seed extracts in biorefinery perspectives.

Nowadays, several extraction, processing, and preservation methodologies have been introduced, with particular regards to green and sustainable techniques for the separation of natural products from waste [36,63].

Examples of non-conventional techniques applied to *Vitis vinifera* waste [64] were given, like enzymatic treatment [65], microwave-assisted extraction [66], ultrasound-assisted extraction [67], and supercritical and subcritical fluid extraction [68,69,70,71,72].

The earliest works about laboratory extraction with supercritical CO_2_, applied to grape byproducts such as skin, seeds, and stems [69], were followed by industrial-scale studies, with multipurpose biorefineries in which polyphenols and sugars extraction in supercritical CO_2_ is accompanied by further processes for the recovery of fatty acids and the production of biogas [70]. Prado et al. [71] reviewed the supercritical fluid extraction of grape seed by describing process scale-up, chemical composition of extracts, and economic evaluation.

The food industry has recently utilized the ultrasound assisted extraction (UAE) process to extract bioactive compounds from plant and animal materials (e.g., polyphenolics, anthocyanins, aromatic compounds, polysaccharides, and functional compounds). This procedure increased the yields of extracted components, rates of extraction, and processing throughput. The optimization of this technology, which complements current methods, could allow for: modification of plant cell material to improve the bioavailability of micronutrients while retaining the natural-like quality; simultaneous extraction and encapsulation; quenching of radical sonochemistry, especially in aqueous systems; avoiding degradation of labile compounds; potential use of radical sonochemistry to achieve targeted hydroxylation of polyphenolics and carotenoids and increase bioactivity [67].

Boussetta and colleagues [73] tested the effects of high-voltage electrical discharges (HVED) on the aqueous extraction of polyphenols from grape pomace at constant temperature in the range of 20–60 °C; HVED increased the extraction kinetics of total solutes and total polyphenols from grape pomace—whatever the method of conservation (fresh, sulphured and frozen)—by damaging both cell membranes and cell walls. The final yields of solutes, reached after HVED application followed by diffusion for 40 min, were more than two-fold higher than the values obtained after 240 min of conventional extraction under similar conditions. Thus, the main advantages of HVED application were the reduced extraction times and temperatures.

Pilot plant scale enzyme-assisted extraction of polyphenols from winery byproducts was optimized after preliminary laboratory tests [74]. Pectinolytic and cellulolytic enzymatic treatment of grape pomace causes the liquefaction of grape skins; this procedure enhances both the extraction yield of polyphenolic compounds and the extraction rates of flavonoids and stilbenes, in respect of those of sulfite extraction. Pre-extraction of pomace with hot water further increased yields of phenolic compounds [74]. A recent extraction method for recovery of phenols from grape seed was developed by Stambuk et al. [75] by application of pectinase, an example of enzyme-assisted extraction.

Spectroscopic techniques coupled to chemometrics could represent a valid green alternative to conventional methods for determination of bioactive compounds in foods and food waste; numerous advantages are given by the use of spectroscopic techniques, with respect to the conventional ones, e.g., simple sample preparation procedure, and short time for data collection and analysis.

Canbay and Bardakçı [76] have carried out structural analysis of grape seed oil and pulp by FT/IR spectrometry by highlighting peculiar functional groups and modes of vibration of main components. Hanganu et al. [77], using the Principal Component Analysis method to the spectral information, studied the application in authenticity control of grape seed oils from common genuine oils (sunflower, soybean, linseed, and rapeseed).

Recently, the spectroscopic technique has been applied to the study and quantification of bioactive compounds in grape seed. Nogales–Bueno et al. [78] have used near-infrared hyperspectral tools for the screening of extractable polyphenols in red grape skins. Further studies [79,80], by jointly applying ATR-FTIR and Raman spectroscopy to grape seed samples, studied and linked the more important spectral features to phenolic extractability and other attributes in grape skin and grape seed. Therefore, FTIR spectroscopy coupled with chemometrics can represent a valuable tool for monitoring the composition of byproducts, allocating them to the most suitable extraction process.

## 5. A Grape Seed Biorefinery: A Picture of Italian Experience

The conversion of winery waste into energy is a source of benefits for wine producers, able to reduce their energy costs by generating renewable energy. Power can be obtained: via biomass gasifiers that convert grape marc to syngas, which is then fed to an internal combustion engine to drive a generator; via biomass boilers used to supply heat to an absorption chiller for fermenter cooling; and via anaerobic digesters used to produce biogas that is directed to an internal combustion engine to drive a generator. Removing polyphenols from the vegetal material before the conversion into energy could be a further important method to increase yields of power due to the antimicrobial potential of these compounds that could inhibit the fermentation processes, and for obtaining new products environmentally and economically sustainable to place on the market. Methods for the recovery of anthocyanins from winery byproducts using sulfite-containing water are not completely suitable because sulfite cannot be removed quantitatively from the extracts, and pseudoallergic reactions caused by foods with added sulfites have been described [81,82]. Extraction with ecocompatible solvents, mainly water, eventually with low percentages of ethilic alcohol, is a suitable method to recover interesting and important bioactive compounds from vegetal matrices [83,84]. This technique, suitable for integration with other processes within biorefinery plants, is ecologically and economically sustainable, as water does not involve high costs, it can be more easily disposed of, and, above all, it can be recovered almost pure while concentrating the extracts to be reintroduced into the production process [85,86,87].

Experimental processes so far tested on a pilot scale in Italy for grape waste (leaves and skin) are based on aqueous extraction accompanied by additional biochemical or physical processes to enhance the extraction yields [85,86].

**Figure 2:**
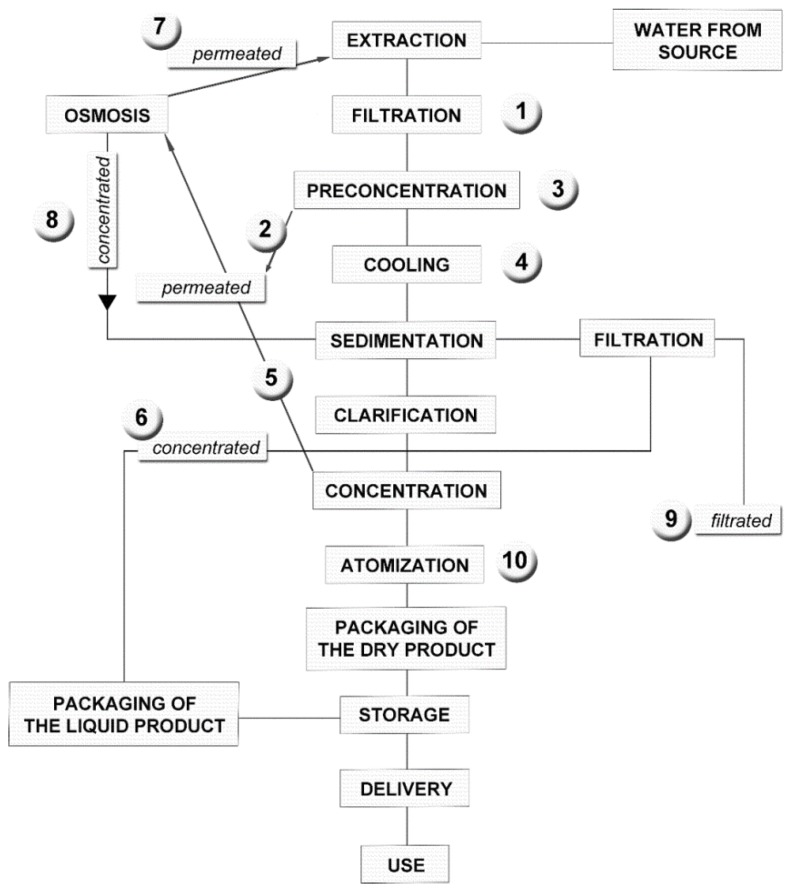
Operating diagram of extraction and fractionation plant from Reference [87], consisting of (**1**) filtered tannin broths; (**2**) permeate from nanofiltration Step 1; (**3**) concentrate from nanofiltration Step 1; (**4**) concentrate from nanofiltration Step 2; (**5**) permeate from nanofiltration Step 2; (**6**) concentrate from nanofiltration Step 3; (**7**) osmosis permeate; (**8**) osmosis concentrate; (**9**) settled fraction from clarification step; (**10**) spray-dried obtained from fraction 6.

An example of an operating diagram for the extraction and purification/concentration of hydrolysable and condensed tannins from vegetal matrices is shown in Figure 2 [87]. The described plant, operating in Tuscany, is suitable for extraction with aqueous or hydroalcoholic solvents, and it can be used to obtain concentrated extracts and fractions of condensed tannins from grape seeds. All the purification and concentration steps are performed by physical processes using semipermeable membranes for micro- and nanofiltration; thus, the whole process can be green and environmentally friendly [85].

The extraction plant works by extracting the vegetal matrices in 100% water or by using low percentages of alcohol. For wood or hard matrices such as grape seeds, yields of aqueous extraction can also be raised by working at high temperatures, compatibly with the stability of the active principles that have to be preserved: in subcritical water extraction processes at high temperatures (150 °C) of grape seeds, there have been observed high yields on procyanidins extraction, but also hydrolysis processes of galloylated compounds, and consequently increased concentrations of free gallic acid [83]. The extraction chamber is fed with 20 m^3^ of biomass and the extraction solvent can reach a temperature of 80 °C. The boiler is fed with the exhausted biomass that has already undergone the polyphenol extraction process, so that the plant can be entirely powered by cogeneration according to the specific process parameters and calorific values of the different matrices (wood matrices have an average calorific value of 2000–5000 Kcal/Kg; the boiler can exploit 10 tons of biomass each batch). The purification and concentration steps are performed by membrane technology, avoiding the use of the organic solvents usually employed in industrial purification processes. This methodology also allows, when necessary, for a selective concentration of specific subclasses of compounds with different chemical and biological properties. The final product is a concentrated extract or spray-dried powder obtained with an average yield of 5% with respect to the vegetal material.

Within the activities of the European project EVERGREEN (Environmentally friendly biomolecules from agricultural wastes as substitutes of pesticides for plant diseases control—LIFE13 ENV/IT/000461) grape seed waste, obtained after wine production and mechanical extraction of oil, was extracted and analyzed for the polyphenol compounds by HPLC/DAD/MS methods [42].

The grape seeds were isolated from marcs obtained after the winemaking process, and pretreated by drying at controlled temperature; the drying parameters were set according to the type of vegetable matrix, with particular attention to avoiding the degradation of the active ingredients. The vegetal material was put in perforated stainless steel baskets inside a drying chamber and maintained at a maximum temperature of 38 °C until its weight was stable. Two batches of seeds pretreatment were monitored in depth: the initial weights were 1260 Kg and 1560 Kg, respectively, and the respective dried weight was 554 Kg and 708 Kg (44% and 45% yields). The dried product was packed in polypropylene bags for food use and stored at room temperature, protected from light.

The dried grape seeds were used for oil mechanical extraction with a 15% yield. The cold-pressing process allows for obtaining totally solvent-free oil, together with an exhausted matrix that consists of the extruded solid after mechanical pressing. The grape seed residue post-oil extraction was considered for agronomical formulations according to the objectives of the Project, since it showed to contain still high levels of polyphenols—in particular condensed tannins.

For this purpose, both seeds and byproducts were characterized by laboratory extraction: for the grape seeds as such, exhaustive extraction in hydroalcoholic solvent (EtOH:H_2_O 70:30, pH 2.5 by HCOOH addition) was performed; for the minced residue after oil extraction, both hydroalcoholic exhaustive extraction and aqueous extraction were performed to obtain respectively a complete characterization and also to reproduce an industrial extraction process with 100% water. The hydroalcoholic extractions confirmed that most of the polyphenolic compounds (72.1%) present in the seeds were retained during the mechanical oil extraction process; thus, the exhausted material could be recovered as a further possibility of exploitation of wine production waste and according to the innovative models of circular agriculture. The measured amounts of single compounds in the aqueous extract of the exhausted residue were as follows (results expressed as mg single compounds per gram of seeds): gallic acid (0.41 mg/g); catechin dimer B3 (1.52 mg/g); catechin (0.64 mg/g); catechin trimer (0.54 mg/g); catechin dimer B6 (0.77 mg/g); catechin dimer B2 (0.96 mg/g); epicatechin (0.59 mg/g); epicatechin gallate dimers I (1.06 mg/g); epicatechin gallate dimers II (5.49 mg/g); catechin tetramers (9.51 mg/g); catechin/epicatechin trimers digallated I (13.47 mg/g); catechin/epicatechin trimers digallated II (8.92 mg/g). Total polyphenols were 43.88 mg/g.

The aqueous extraction of seed waste after oil extraction allowed for a more sustainable, even though lower, recovery of polyphenolic compounds; in this case, an increased percentage of gallic acid was observed in byproducts with respect to the seeds (0.93% vs. 0.04%)—this could be due to partial hydrolysis of heavier molecules.

Recent studies have shown high antioxidant and antimicrobial properties for gallic acid in particular, and higher nematocidal activity for extracts particularly rich in free gallic acid has also been observed [88,89]; thus, it is of interest to test new agronomical formulations containing oil extraction residue from grape seeds: these formulations could slowly release condensed tannins into the ground to obtain antimicrobial and nematostatic/nematicidal effects.

In this order, a new gel formulation containing the minced grape seed residue post-oil extraction was designed, produced, and monitored for 4 months by spectrophotometric analysis repeated at regular time intervals (1 month). The preliminary analyses showed that the powder obtained from the spent residue gradually releases polyphenolic compounds into the gel matrix along with time until flavan-3-ol and procyanidin concentration, expressed as catechin equivalents per gram of gel, was 7.4 mg/g gel (0.7% *p*/*p*).

The formulations, chemically and physically stable within the time of use, are in a test phase on several crops to prevent the attack from various pathogenic species.

## 6. Conclusions

In Italy, the production of waste from winemaking processes is high and its use following a holistic biorefinery approach is needed. The integrated exploitation of enological byproducts represents a basis for an ‘intelligent’ reconversion of waste that can be attained through the use of eco-compatible ‘green technologies’ to ensure environmental sustainability in the supply chain. In this context, studies are needed aimed at optimizing an integrated valorization of biomass, as well as to guide producers and consumers towards virtuous and sustainable development paths.
